# Obituary

**Published:** 1894-02

**Authors:** 


### Obituary.

Dr. Willard C. Marselius died at his residence, in Albany, on Sunday, December 24, 1893. He was taken ill the Wednesday previous with appendicitis, and an operation was performed Friday morning by Dr. Albert Vander Veer, assisted by Drs. Macdonald and Ward. The appendix was found to be gangrenous, and there was intestinal perforation with general peritonitis. The operation consisted in removing the appendix and closing the perforations, and it was borne without serious shock. The inflammation, however, extended, and within forty-eight hours death ensued.

Dr. Marselius was born in Schenectady county, and was descended from one of the oldest Dutch families in the State. He was a graduate of Union University, class of '81, and in 1884 he was graduated from Albany Medical College. In 1886, he became associated in the practice of medicine with his uncle, Dr. Vander Veer, which relation continued until his death. He was one of the representative physicians of Albany, and was esteemed by both profession and laity. He was married, September 12, 1893, to Miss Gertrude E. Wheeler, of Massachusetts, whose bereavement appeals to the sympathy of a wide circle of friends.

---

Dr. Herbert Judd, of Galesburg, Ill., died suddenly, from apoplexy, at his residence, January 11, 1894, aged 50 years. He graduated at the Albany Medical College in 1867, and has practised his profession in Galesburg since that time. Dr. Judd had been surgeon of the Chicago, Burlington & Quincy Railway and at the time of his death, as well as for some years previous thereto, was surgeon of the Chicago, Santa Fé & California Railway. He was a surgeon of eminence and a physician who practised his profession with a full measure of success. In the city of his residence he was regarded as a foremost man of affairs, and had a large acquaintanceship throughout the United States that will be pained to learn of his sudden and early death.

